# Transcriptome analysis reveals differentially expressed genes between human primary bone marrow mesenchymal stem cells and human primary dermal fibroblasts

**DOI:** 10.3906/biy-1808-81

**Published:** 2019-02-07

**Authors:** Ekim Zihni TAŞKIRAN, Beren KARAOSMANOĞLU

**Affiliations:** 1 Department of Medical Genetics, Faculty of Medicine, Hacettepe University , Ankara , Turkey

**Keywords:** Human bone marrow mesenchymal stem/stromal cells, human dermal fibroblasts, transcriptomics, gene expression

## Abstract

Stromal cells have been widely used in biomedical research and disease modeling studies in vitro. The most commonly used types of stromal cells are mesenchymal stem cells and fibroblasts. Their cellular phenotypes and differentiation capabilities are quite similar and there are no specific distinction criteria. In order to identify transcriptomic differences between these 2 cell types, we performed next-generation RNA sequencing. Using the global gene expression profile and pathway analysis, we showed that human primary bone marrow mesenchymal stem cells and human primary dermal fibroblasts have different molecular signatures. We also identified critical transcription factors that are differentially expressed between these cells. We then proposed that homeobox genes and some other sequence-specific transcription factors are not only responsible for transcriptional differences, but also discriminate bone marrow mesenchymal stem cells and dermal fibroblasts at the transcriptional level.

## 1. Introduction

Stromal cells have been widely used in biomedical research and disease modeling studies in vitro. One of the most important types of stromal cells is the mesenchymal stem/stromal cell (MSC). MSCs are multipotent stem cells that originate from mesenchyme and can be isolated from many tissues, but mainly from bone marrow and adipose tissues (Charbord, 2010; Hass, 2011) . Bone marrow mesenchymal stem/stromal cells (BM-MSCs) are positive for specific surface markers CD90, CD73, and CD105 and negative for specific surface markers CD45, CD34, CD14, and CD19 according to the International Society for Cellular Therapy (ISCT) (Bae, 2009; Charbord, 2010; Pontikoglou, 2011) . They are spindle-shaped adherent cells and have the capacity to differentiate into cell types of this lineage such as adipocyte, osteocyte, chondrocyte, myocyte, tendocyte, and ligamentocyte (Pontikoglou, 2011) . They are one of the most important members of bone marrow and responsible for stromal support. In addition, they are capable of migrating into damaged tissue and play a major role in repair or regeneration of that tissue. They have high regeneration potential and immune modulatory properties (Charbord, 2010; Eggenhofer, 2014) . These features make MSCs very advantageous for regenerative medicine and therapeutic approaches.

Another type of stromal cell is the dermal fibroblast (DF), which may be obtained easily from skin biopsy or different surgical materials. DFs are mainly responsible for extracellular matrix (ECM) synthesis; they are found in the dermal layer of the skin. Fibroblasts that are derived from this layer are responsible for forming connective tissue and play a major role in wound healing or repair at injury sites (Driskell, 2015) . Thus, they provide healing and recovery in almost all tissues. However, activation and proliferation of fibroblasts sometimes lead to bfirosis or scar formation (Driskell, 2013) . BM-MSCs and DFs may not be distinguished by their cellular morphology or surface markers (Bae, 2009; Denu, 2016, Ichim, 2018; Kundrotas, 2012; Soundararajan, 2018) . Due to their common properties, there should be a detailed study in order to reveal their differential characteristics. Transcriptomic studies are highly important for evaluating cell-specific characteristics (Kasoju, 2017) . The aim of this study is to reveal the transcriptomic profiles of human BM-MSCs and DFs in order to identify discriminating markers.

## 2. Materials and methods

### 2.1. Cell culture and RNA isolation

Human primary bone marrow mesenchymal stem/stromal cells (BM-MSCs) (Cat. No: PCS-500-012™) and Human primary dermal fibroblasts (DFs) (Cat. No: PCS-201-012™) were purchased from American Type Culture Collection (ATCC, Manassas, VA, USA). BM-MSCs were obtained from a 24-year-old male Caucasian donor’s bone marrow aspirate (Lot: 63208778) and DFs were obtained from a 28-year-old male African–American donor’s abdominal skin (Lot: 63792061). Cells were incubated at 37 °C, 5% CO2 conditions; passage 3 cells were used, and all samples were prepared in triplicate. Culture medium (DMEM-LG, 10% FBS, 1% Pen/Strep, 1% L-glutamine) was changed twice a week. Aeftr reaching nearly 70%–80% confluency, cells were washed with PBS and treated with TRI Reagent® for RNA isolation (Sigma, St. Louis, MO, USA). RNA was isolated according to the manufacturer’s instructions. RNA quality was measured with a Nanodrop® (Thermo Fisher Scientific, Waltham, MA, USA) spectrophotometer and quantity was measured with a Qubit® (Thermo Fisher Scientific) flourometer.

### 2.2. Quantitative transcriptome analysis

For library preparation, a barcoded cDNA library was first generated with a SuperScript VILO cDNA Synthesis Kit (Thermo Fisher Scientific) from 10  ng of total RNA sample. An Ampliseq Human Gene Expression Chef Ready Kit (Thermo Fisher Scientific) was used for targeted amplification of nearly 20,000 distinct mRNA targets. Libraries were then generated by an Ion Chef instrument (Thermo Fisher Scientific), and pooled libraries were clonally amplified using emulsion PCR on an Ion Torrent OneTouch2 (OT2) instrument (Thermo Fisher Scientific). Enrichment was done using an Ion OneTouch ES instrument (Thermo Fisher Scientific). The templated libraries were then sequenced on an Ion Proton semiconductor sequencing system, using an Ion PI Hi-Q Sequencing Kit and Ion PI chip v3 (Thermo Fisher Scientific). All next-generation sequencing experiments were performed in duplicate.

### 2.3. Data analysis

The sequencing data were processed by the Torrent Suite analysis pipeline. Raw reads were mapped to the human genome assembly hg19 AmpliSeq Transcriptome version by TMAP (Torrent mapping alignment program). The data were normalized and differentially expressed genes (DEG) were determined with DESeq2. STRING (version 10.5) was used to identify associations between the significant genes (http://string-db.org/).

## 3. Results

**Figure 1:**
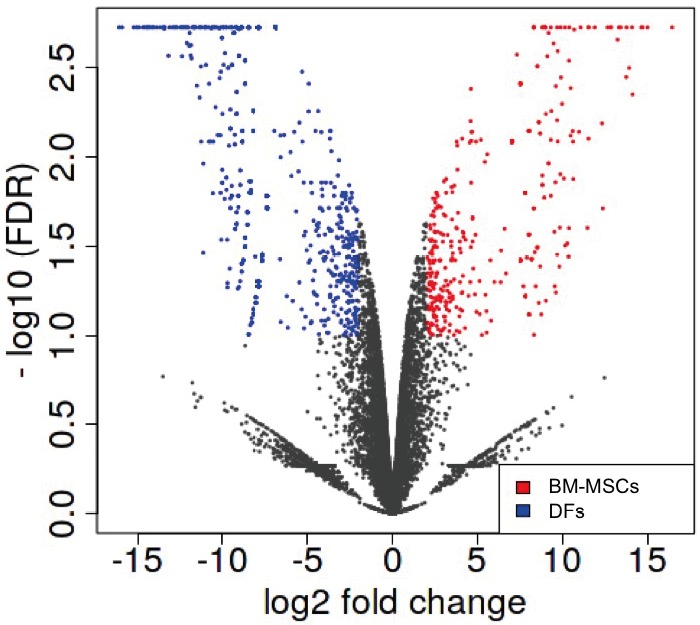
Volcano plot of differentially expressed genes between BM-MSCs (red) and DFs (blue) (log_2_ fold change versus –log_10_ false discovery rates).

We found that 419 and 773 genes were upregulated in Human Primary BM-MSCs and in Human Primary DFs, respectively. Volcano plot indicated that these gene expression changes were statistically significant (Figure 1). We then continued further analysis with these differentially expressed genes (DEGs).

**Figure 2:**
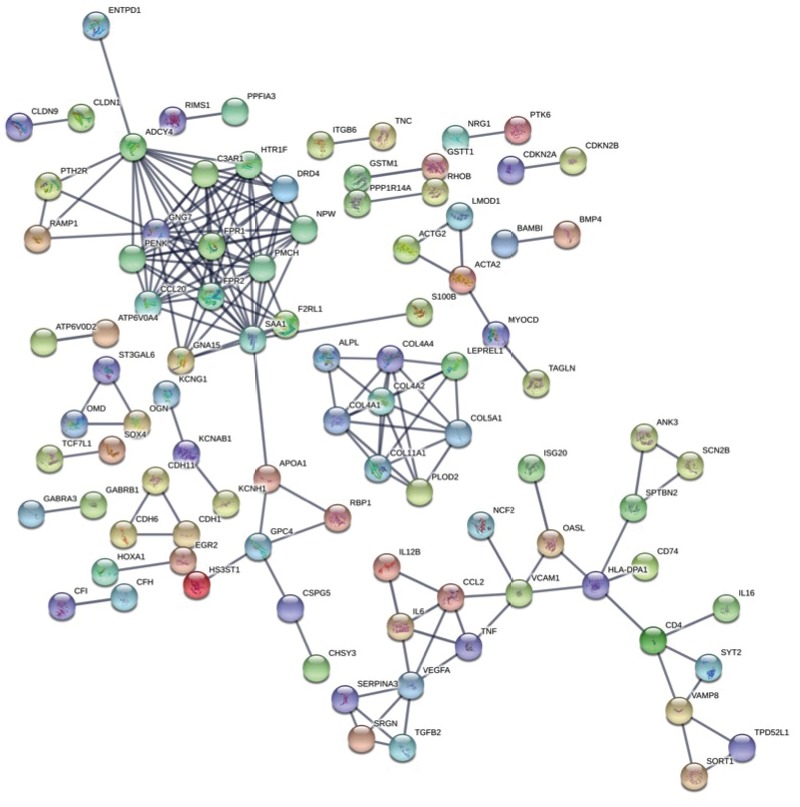
STRING output of network analysis of the DEGs in BM-MSCs (confidence score = 0.900).

We performed STRING analysis for protein interactions and connections with upregulated genes in BM-MSCs. We found higher interaction between immune-system– related genes (IL6, VEGFA, TNF, TGFB2, CCL2, IL12B, HLA-DPA1, CD4, IL16, SAA1, FPR1, FPR2, CFI, and CFH) in BM-MSCs (Figure 2). Pathway analysis showed that most of the DEGs in BM-MSCs were significantly enriched regarding receptor binding, sequence-specific DNA binding, protein binding, growth factor activity, transcriptional activator activity, and extracellular matrix structural constituent (Table 1).

**Table 1:** DEGs in BM-MSCs annotated using gene ontology molecular function.

Pathway ID	Pathway description	Observed gene count	False discovery rate
GO:0005102	Receptor binding	48	2.5e-05
GO:0043565	Sequence-specific DNA binding	40	3.85e-05
GO:0005515	Protein binding	117	0.00511
GO:0008083	Growth factor activity	9	0.00856
GO:0001228	Transcriptional activator activity, RNA polymerase II transcription regulatory region sequence-specific binding	18	0.0121
GO:0000976	Transcription regulatory region sequence-specific DNA binding	24	0.0137
GO:0005201	Extracellular matrix structural constituent	8	0.0137
GO:1901681	Sulfur compound binding	14	0.0137
GO:0000982	Transcription factor activity, RNA polymerase II core promoter proximal region sequence-specific binding	17	0.0252
GO:0001077	Transcriptional activator activity, RNA polymerase II core promoter proximal region sequence-specific binding	14	0.0252
GO:0005539	Glycosaminoglycan binding	13	0.0252
GO:0008201	Heparin binding	11	0.0252
GO:0044212	Transcription regulatory region DNA binding	26	0.0252
GO:0005126	Cytokine receptor binding	13	0.0335
GO:0015077	Monovalent inorganic cation transmembrane transporter activity	17	0.0431
GO:0005261	Cation channel activity	15	0.0436
GO:0070851	Growth factor receptor binding	8	0.0436

**Figure 3:**
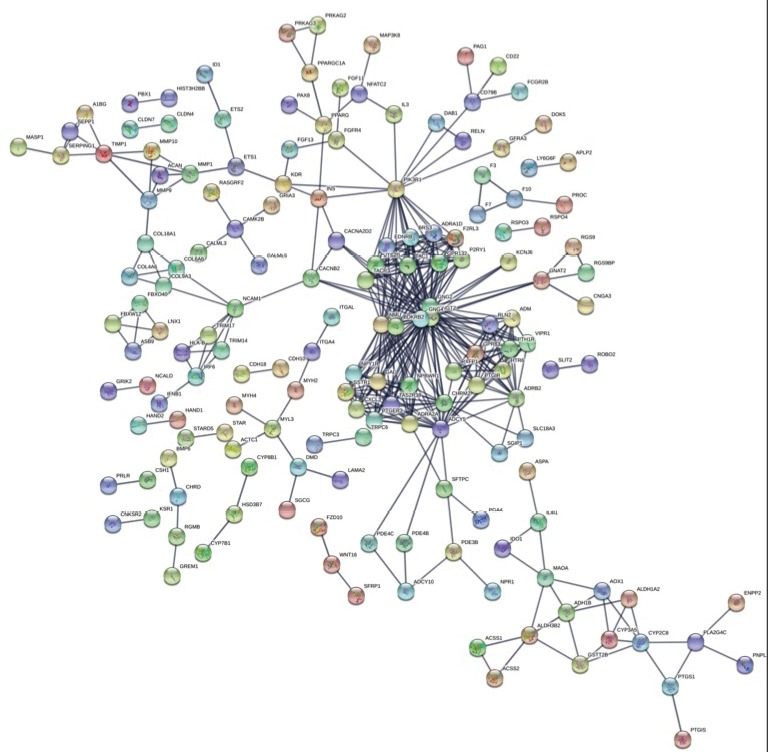
STRING output of network analysis of the DEGs in DFs (confidence score = 0.900).

STRING analysis showed that DEGs in DFs were also closely related (Figure 3). According to pathway analysis of DEGs in DFs were significantly enriched regarding transmembrane signaling receptor activity, signaling receptor activity, signal transducer activity, receptor activity, molecular transducer activity, and G-protein coupled receptor activity and calcium ion binding (Table 2).

**Table 2:** DEGs in DFs annotated using gene ontology molecular function.

Pathway ID	Pathway description	Observed gene count	False discovery rate
GO:0004888	Transmembrane signaling receptor activity	81	2.9e-09
GO:0038023	Signaling receptor activity	85	2.9e-09
GO:0004871	Signal transducer activity	98	3.58e-09
GO:0004872	Receptor activity	92	3.58e-09
GO:0060089	Molecular transducer activity	105	4.03e-09
GO:0004930	G-protein coupled receptor activity	57	1.35e-06
GO:0005509	Calcium ion binding	44	0.00503

**Figure 4:**
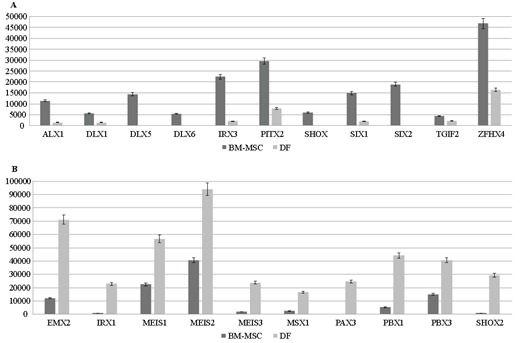
Differentially expressed homeobox genes in BM-MSCs (A) and DFs (B). The y-axis represents normalized read counts.

**Figure 5:**
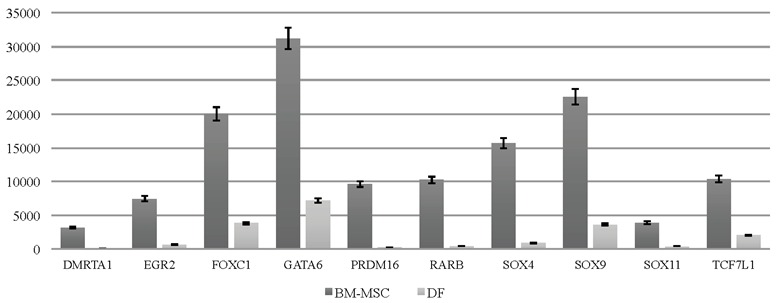
Transcription factors predominantly expressed in BM-MSCs. The y-axis represents normalized read counts.

Becausesequence-specific DNA binding proteins showed different expression patterns between BM-MSCs and DFs, we first examined all of the probable functional homeobox genes in detail (Holland, 2007) . In this examination, homeobox genes showed cell-specific expression pattern. ALX1, DLX1, DLX5, DLX6, IRX3, PITX2, SHOX, SIX1, SIX2, and ZFHX4 were highly expressed in BM-MSCs, while EMX2, IRX1, MEIS1, MEIS2, MEIS3, MSX1, PAX3, PBX1, PBX3, and SHOX2 were highly expressed in DFs (Figures 4A and 4B). In addition to homeobox genes, we found that some of the other transcription factors that are important for several biological functions were highly expressed in BM-MSCs (Figure 5).

## 4. Discussion

To date, there have been many studies on the similarities and differences between MSCs and DFs (Bae, 2009; Ichim, 2018; Kundrotas, 2012; Ulrich, 2012; Soundararajan, 2018) . However, there is no agreement about the exact discrimination of these cells yet. Previous studies about transcriptomic profiling of stromal cells were accomplished with microarray-based techniques (Bae, 2009) . Herein, we performed up-to-date next-generation sequencing technology to quantify transcriptomic differences. By using next generation RNA-sequencing we obtained more detailed data including even low-level transcripts (Li, 2015) . We first identified differentially expressed genes between Human Primary BM-MSCs and Human Primary DFs, and then focused on the expression levels of some transcription factors which could be responsible for gene expression differences between these 2 cell types. Comparative analysis showed that BM-MSCs and DFs are distinguishable according to their global gene expression profile.

We observed that homeobox genes were differentially expressed between BM-MSCs and DFs. The homeobox genes are a large group of genes which play important roles in embryonic development (Seifert, 2015) . They encode transcription factors regulating cellular processes such as proliferation and migration. According to our analysis, DLX1, DLX5, and DLX6 were predominantly expressed in BM-MSCs. It has been shown in recent studies that some members of the DLX (distal-less homeobox) family are responsible for osteogenic differentiation and craniofacial development ( Charite, 2001 ; Li, 2008 ; Heo, 2017 ). The aristaless-like homeobox family member ALX1, which is necessary for the development of the head and face, is also more highly expressed in BM-MSCs than in DFs (Zhao, 1996) . Another highly expressed homeobox gene in BMMSCs is SHOX, which regulates the expression of early osteogenic genes during differentiation (Yokokura, 2017) . When all of these are taken into account, one could think that for studying developmental gene expression networks and osteogenic differentiation stages, BM-MSCs are more appropriate than DFs.

The predominantly expressed homeobox genes in DFs mainly belong to the TALE (three amino acid loop extension) homeobox family (IRX1, MEIS1, MEIS2, MEIS3, and PBX1). Unlike other genes within homeobox genes, TALE group members are widely expressed rather than cell/tissue-specific (Dunwell, 2016) . The stromal cellspecific functions of these have not yet been reported. However, due to the presence of studies that link bfirosis with some homeobox genes, their role in this condition should be investigated in detail (Wandzioch, 2004; Zhou, 2014; Gong, 2017) .

There are also some critical targets, predominantly expressed in BM-MSCs, which could be the basis of gene expression differences between these 2 cell types. According to recent publications, most of the differentially expressed transcription factors have critical roles in stem cell biology. For example, it has been shown that SOX family members are responsible for cell fate decisions, and it has also been shown that SOX11, GATA6, and PRDM16 have critical roles in MSC self-renewal (Kubo, 2009; Sarkar, 2013) . Additionally, FOXC1, highly expressed in BM-MSCs, is identified as a key player in the bone marrow niche (Omatsu, 2014) . When we tried to identify the master regulator of immune-system–related genes in our data, we showed that EGR2 (early growth response IRX32) transcription factor was highly expressed in BM-MSCs but not in DFs. It is known that EGR2 is responsible for mediating the expression of immune-system–related genes in MSCs (Barbeau, 2014) . We proposed that expression levels of all these transcription factors could be used as discriminating markers between BM-MSCs and DFs.

This is an example of RNA-sequencing–based transcriptomics regarding comparison of different stromal cells. Future studies should focus on stage- and/or diseasespecific transcriptomic signatures between different types of stromal cells. By doing this, biological functions and therapeutic potentials of stromal cells could be identified in detail.
